# Enzyme Cascade Amplification-Based Immunoassay Using Alkaline Phosphatase-Linked Single-Chain Variable Fragment Fusion Tracer and MnO_2_ Nanosheets for Detection of Deoxynivalenol in Corn Samples

**DOI:** 10.3390/foods13132009

**Published:** 2024-06-25

**Authors:** Guifang Xie, Fujing Mao, Yirui Huang, Li Wen, Zhichang Sun, Zhenyun He, Xing Liu

**Affiliations:** 1 School of Food Science and Engineering, Hainan University, Haikou 570228, China; 13208910510@163.com (G.X.); maofujing11@163.com (F.M.); nancyeer@hainanu.edu.cn (Y.H.); wenli13765447429@163.com (L.W.); sunzhichang11@163.com (Z.S.); 2 Guizhou Provincial Supervision and Testing Center for Agricultural Product Quality, Agricultural Product Quality and Safety Risk Assessment Laboratory of the Ministry of Agriculture, Guiyang 550004, China; 3 School of International Tourism, Hainan College of Economics and Business, Haikou 571127, China

**Keywords:** mycotoxin, single-chain fragment variable, alkaline phosphatase, MnO_2_ nanosheets, immunoassay, cereal

## Abstract

Deoxynivalenol (DON) is a common mycotoxin that contaminates cereals. Therefore, the development of sensitive and efficient detection methods for DON is essential to guarantee food safety and human health. In this study, an enzyme cascade amplification-based immunoassay (ECAIA) using a dual-functional alkaline phosphatase-linked single-chain fragment variable fusion tracer (scFv-ALP) and MnO_2_ nanosheets was established for DON detection. The scFv-ALP effectively catalyzes the hydrolysis of ascorbyl-2-phosphate (AAP) to produce ascorbic acid (AA). This AA subsequently interacts with MnO_2_ nanosheets to initiate a redox reaction that results in the loss of oxidizing properties of MnO_2_. In the absence of ALP, MnO_2_ nanosheets can oxidize 3,3′,5,5′-tetramethylbenzidine (TMB) to produce the blue oxidized product of TMB, which exhibits a signal at a wavelength of 650 nm for quantitative analysis. After optimization, the ECAIA had a limit of detection of 0.45 ng/mL and a linear range of 1.2–35.41 ng/mL. The ECAIA exhibited good accuracy in recovery experiments and high selectivity for DON. Moreover, the detection results of the actual corn samples correlated well with those from high-performance liquid chromatography. Overall, the proposed ECAIA based on the scFv-ALP and MnO_2_ nanosheets was demonstrated as a reliable tool for the detection of DON in corn samples.

## 1. Introduction

Deoxynivalenol (DON) is a secondary metabolite produced by *Fusarium graminearum* [1]. Due to the prevalence of toxin-producing strains in warm and humid environments, DON contamination is widespread in stored crops, feed, and foodstuffs [2]. Studies have shown that DON can inhibit animal protein synthesis, damage immune cells, severely impact the immune system, and potentially lead to cancer [3,4]. To address the serious health risks associated with DON, many countries and organizations have established limits on its presence in food [5]. For instance, the Codex Alimentarius Commission (CAC) has instituted DON limits of 2000 μg/kg for corn, barley, and wheat, and 1000 μg/kg for derivative cereal products. In China, the regulatory threshold for grains and their derivatives stands at 1000 μg/kg. DON exhibits excellent thermal stability, surviving high-temperature cooking and baking processes in contaminated food [6]. Therefore, it is crucial to establish sensitive and efficient detection methods to ensure food safety and minimize economic losses.

Currently, instrumental analysis methods are widely used for the detection of exogenous contaminants such as mycotoxins, pesticides, and veterinary drugs [7,8,9]. However, the complex sample preparation processes and high costs associated with these methods have spurred the development of immunoassay methods. Immunoassay methods mainly include enzyme-linked immunosorbent assay (ELISA), electrochemical immunoassay, chemiluminescent immunoassay, fluorescent immunoassay, lateral flow immunoassay, and microfluidic-based immunoassay [10,11,12,13,14,15,16,17,18,19]. Although these immunoassays differ in their detection formats, they all rely on the specific binding between antigens and antibodies. Despite the common use of monoclonal antibodies in immunoassays, limitations in terms of high cost and complex preparation steps have hindered their further applications [20,21].

The emergence of genetically engineered recombinant antibodies overcomes the limitations of traditional intact antibodies [22,23,24]. With the development of phage display technology, genetically engineered antibodies can be produced through simplified and quantitative biopanning, replacing the traditional complex hybridoma technology [21,25]. Research has shown that genetically engineered recombinant antibodies have been successfully expressed in prokaryotic and eukaryotic cells [26,27]. Among them, the single-chain fragment variable (scFv) has been very attractive because of its small size and low production cost [25,28]. Moreover, the significant advantage of the scFv is its ease of genetic manipulation [29], which contributes to the generation of many bifunctional fusion proteins [30,31]. In this study, the scFv was fused with alkaline phosphatase (scFv-ALP) for expression, enabling the protein to possess both the antibody’s specific recognition and the enzyme’s catalytic amplification ability. Despite their high catalytic efficiency and specificity, protein-based conventional biocatalysts are often characterized by poor stability and susceptibility to environmental influences, thereby significantly increasing storage costs [32].

Advancements in nanotechnology have led to the exploration of “nanozymes” that simulate the catalytic functions of traditional biological enzymes [32,33]. For instance, Yan’s team first demonstrated the peroxidase-like activity of iron oxide (Fe_3_O_4_) nanoparticles and applied it to immunoassays [34]. Compared to biological enzymes, nanozymes are more stable, efficient, and capable of large-scale production [35]. In simple terms, nanozymes can be classified into oxidoreductases and hydrolases based on their catalytic principles. For example, metal-based, carbon-based, and metal–organic framework nanozymes have been widely used in food safety testing [32]. Among them, MnO_2_ nanosheets are a type of metal-based nanozyme with peroxidase-like properties. Its unique two-dimensional (2D) structure provides a large surface area, enabling efficient catalytic performance [36]. Similar to horseradish peroxidase (HRP), MnO_2_ nanosheets can oxidize 3,3′,5,5′-tetramethylbenzidine (TMB) to the blue oxidized product of TMB (oxTMB). Additionally, research has found that ascorbic acid or glutathione can reduce MnO_2_ nanosheets to Mn^2+^ [36,37]. Therefore, the catalytic properties of nanozymes render them applicable in rapid detection for food safety.

In this study, to expand the application of the scFv-ALP in immunoassays, an enzyme cascade amplification immunoassay (ECAIA) for deoxynivalenol (DON) was developed using the scFv-ALP and MnO_2_ nanosheets. The construction of the MnO_2_ nanosheets and TMB-based (MnO_2_-TMB) sensing system and its feasibility for detecting the scFv-ALP were described in detail. By integrating the scFv-ALP-based immunoassay system with the MnO_2_-TMB sensing system, the ECAIA can implement the detection of DON in food. The optimization of experimental parameters and the methodological evaluation of ECAIA were described in detail.

## 2. Materials and Methods

### 2.1. Material and Reagents

Tetramethylammonium hydroxide (TMA·OH) and manganese chloride tetrahydrate (MnCl_4_·H_2_O) were purchased from Aladdin (Shanghai, China). Ascorbic acid (AA) and ascorbyl 2-phosphate (AAP) were obtained from Jinming Biotech (Beijing, China). In addition, 96-well microplates and 3,3′,5,5′-tetramethylbenzidine (TMB) were purchased from Sangon Biotech (Shanghai, China). The standards of DON, fumonisin B_1_ (FB_1_), zearalenone (ZEN), ochratoxin A (OTA), and aflatoxin B_1_ (AFB_1_) were procured from Pribolab (Qingdao, China). The artificial antigen DON-bovine serum albumin conjugate (DON-BSA) was obtained from Green Valley Biotech (Shandong, China). The nickel−nitrilotriacetic acid agarose gel (Ni-NTA) was purchased from Solabio (Beijing, China). The HRP conjugated anti-his tag mouse monoclonal antibody was obtained from CoWin Biosciences (Taizhou, China). The engineered *E. coli* BL21(DE3) strain containing the recombinant vector pET25b-scFv-ALP for the anti-DON fusion tracer scFv-ALP was previously constructed in our laboratory [38]. All chemicals and reagents are of analytical grade or chromatographic grade as required.

### 2.2. Expression, Purification, and Characterization of the scFv-ALP Fusion Tracer

The engineered *E. coli* BL21(DE3) strain was inoculated into the culture medium for the auto-induction expression of the scFv-ALP fusion tracer as described previously [39]. After auto-induction expression, the bacterial cells from 200 mL culture were collected by centrifugation and resuspended in 10 mL of Tris-buffered saline (TBS, 50 mM, pH 7.4) for sonication. After centrifugation, the supernatant was transferred onto a column (1.6 × 2.5 cm) preloaded with Ni-NTA agarose for purification and elution according to the manufacturer’s manual. The eluted protein was dialyzed in TBS at 4 °C for 72 h and then quantified using a Micro Drop ultramicro UV spectrophotometer (BIO-DL Corporation, Shanghai, China). Further validation on the expression and purification of the fusion tracer was performed using sodium dodecyl sulfate-polyacrylamide gel electrophoresis (SDS-PAGE) and Western blot as described previously [40]. The purified fusion tracer was aliquoted for frozen storage at −20 °C before use.

To validate the functionality of the fusion tracer, the activities of both the antibody and enzyme were tested. Firstly, the antibody activity was assessed by an indirect ELISA as follows. Briefly, a 96-well microplate was coated with 100 μL/well of DON-BSA (2 μg/mL in TBS) at 37 °C for 2 h and blocked with 300 μL/well of 3% (*m*/*v*) skim milk powder in TBS at 37 °C for 1 h. After washing three times using the TBS with 0.05% (*v*/*v*) Tween-20 (TBST), the microplate was incubated with 100 μL/well of the scFv-ALP with various concentrations at room temperature for 1 h. Then, 100 μL/well of HRP conjugated anti-his tag mouse monoclonal antibody (0.33 μg/mL in TBS) was added and incubated at 37 °C for 1 h. After washing four times with TBST, the microplate was incubated with 100 μL/well of TMB substrate solution for color reaction (37 °C, 10 min). The reaction was terminated by adding 50 μL/well of 2 M H_2_SO_4_. The absorbance at 450 nm (OD_450_) of the yellow products was measured using a microplate reader (ST-360, Shanghai Kehua Bio-Engineering Co., Ltd., Shanghai, China). The ΔA value was calculated using the formula ΔA = (OD_450_ of MP-ALP) − (OD_450_ of the blank well). A curve of ΔA versus the scFv-ALP concentration was plotted. Secondly, the enzyme activity was evaluated using a similar procedure, except that the scFv-ALP was not incubated with the secondary antibody. Instead, 100 μL of 0.1% (*w*/*v*) pNPP-diethanolamine buffer was added to each well, and the plate was incubated in the dark for 10 min. The ΔA value was calculated using the formula ΔA = (OD_405_ of scFv-ALP) − (OD_405_ of the blank well). A curve of ΔA versus the scFv-ALP concentration was plotted.

### 2.3. Synthesis and Characterization of MnO_2_ Nanosheets

The MnO_2_ nanosheets were synthesized as described previously with minor modifications [41]. Briefly, a mixture containing 20 mL of 0.6 M TMA·OH and 2 mL of 30% H_2_O_2_ was added to 10 mL of 0.3 M MnCl_4_·solution. After vigorous stirring at room temperature for 12 h, the mixed solution was centrifugated (10,000× *g*, 10 min) to collect the precipitate. Subsequently, the precipitate was washed three times with ultrapure water and anhydrous ethanol, followed by drying at 60 °C. The dried precipitate was dispersed in ultrapure water by sonication for 16 h. The un-separated MnO_2_ nanosheets were removed by centrifugation (600× *g*, 10 min), and the supernatant was transferred for another centrifugation (800× *g*, 10 min) before storage at 4 °C. The concentration of the as-prepared MnO_2_ nanosheets in the aqueous solution was determined by the Lambert–Beer law, with a molar extinction coefficient of 9.6 × 10^3^ M^−1^cm^−1^ at 480 nm. The characterization of the MnO_2_ nanosheets was performed using JEM 2100 transmission electron microscopy (TEM) (JEOL Ltd., Tokyo, Japan), Bruker Dimension Edge atomic force microscopy (AFM) (Bruker Corporation, Billerica, MA, USA), and Malvern Zetasizer Nano ZS90 (Malvern Instruments Ltd., Malvern, UK). The UV–visible absorption spectra and absorbance were obtained using a spectral scanning multi-mode reader SP-Max 3500FL (Flash Spectrum Inc., Shanghai, China).

### 2.4. Enzyme Cascade Amplification-Based Immunoassay for DON

Based on the dual-functional fusion protein scFv-ALP and MnO_2_ nanosheets, an enzyme cascade amplification-based immunoassay (ECAIA) for detecting DON was constructed as follows. First, a 96-well microplate was pretreated and blocked as described in Section 2.2. Then, 50 μL of a series of DON standard solutions and 50 μL of the scFv-ALP (10 μg/mL in TBS) were added into the microplate and incubated at 37 °C for 1 h. After each incubation step, the microplate was washed three times with TBST. Subsequently, the microplate was incubated with 40 μL of 250 μM AAP, 10 μL of MnO_2_ nanosheets solution (1 mM), and 100 μL of NaAc-Hac buffer (20 mM NaAc, 20 mM HAc, pH adjusted to 3.8) at 37 °C for 1 h. Finally, 100 μL/well of TMB substrate solution was added for incubation in the dark at 37 °C for 10 min, and the absorbance at 650 nm (OD_650_) for each well was measured using a spectral scanning multi-mode reader SP-Max 3500FL (Flash Spectrum Inc., Shanghai, China). For the quantitative analysis of DON, a standard competitive inhibition curve was established by plotting the logarithm of DON concentration against the percentage binding rate using a four-parameter logistic equation of Origin2019 (OriginLab Corporation, Northampton, MA, USA). The percentage binding rate was calculated using the following formula: binding rate (%) = (A − A_blank_)/(A_0_ − A_blank_) × 100%, where A is the absorbance in the presence of DON and scFv-ALP, A_blank_ is the absorbance of blank without DON and scFv-ALP, and A_0_ is the absorbance in the presence of the scFv-ALP and without DON. The experimental results were analyzed and judged based on the half-maximal inhibitory concentration (IC_50_).

### 2.5. Selectivity of the ECAIA for DON

To validate the selectivity of the developed ECAIA, four common cereal mycotoxins including AFB_1_, OTA, ZEN, and FB_1_ were used to replace DON for cross-reaction analysis. The percentage cross-reactivity (CR) rate was calculated as follows: CR (%) = [IC_50_ (substitute)/IC_50_ (DON)] × 100%.

### 2.6. Sample Analysis and Validation

The corn samples stored in the lab were pretreated and analyzed as described below. First, 1 g of the ground cereal sample was accurately weighed into a 15 mL centrifugal tube with 4 mL of TBS. The sample was extracted by vigorously shaking on a horizontal shaker for 20 min. After centrifugation, the supernatant was transferred and diluted properly with TBS for ECAIA analysis. For the spike-and-recovery experiment, the negative samples validated by HPLC with an ultraviolet detector (HPLC-UVD) were spiked with various levels of DON standard, followed by extraction and measurement as described above. To further verify the effectiveness of the proposed method for DON, the authentic cereal samples were synchronously pretreated for analysis by HPLC-UVD (Table S1) according to the national standard GB5009.111-2016 of China [42] with minor modifications, as described in the Supplementary Materials.

## 3. Results and Discussion

### 3.1. Expression, Purification, and Characterization of the scFv-ALP

**Figure 1:**
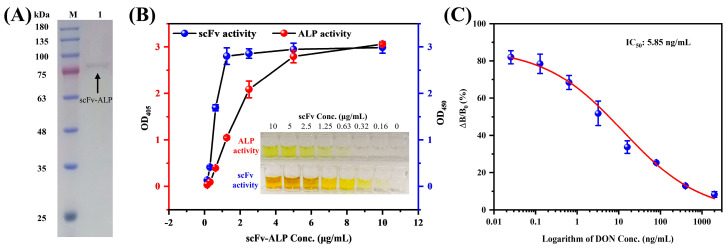
Characterization of fusion protein scFv-ALP. (**A**) SDS-PAGE analysis of the purified scFv-ALP. Lane M: prestained protein marker; lane 1: purified scFv-ALP with a molecular weight of 81 kDa. (**B**) Analysis of the enzymatic activity and antibody functionality within scFv-ALP. Inset: diagrams of the reaction wells of the 96-well microplate. (**C**) Analysis of the detection performance of scFv-ALP by the direct competitive ELISA. Error bars denote the standard deviations (SDs) of three independent experiments.

The bifunctional fusion protein scFv-ALP was obtained by auto-induced expression in the engineered *E. coli* BL21(DE3) strain containing the recombinant plasmid pET25b-scFv-ALP. The purified protein was characterized using SDS-PAGE. As shown in Figure 1A, a predicted single clear band appeared at around 81 kDa, which corresponds to the calculated molecular weight of the scFv-ALP based on its nucleotide sequence. Moreover, the result indicated that the target protein scFv-ALP with high purity was successfully prepared. Subsequently, we further investigated whether both scFv and ALP maintained their activity in the dual-functional fusion protein scFv-ALP. As shown in Figure 1B, the OD_405_ and OD_450_ values increased and reached a plateau as the concentration of the scFv-ALP fusion tracer increased from 0.16 μg/mL to 10 μg/mL. Therefore, after prokaryotic expression, both the scFv and ALP of the fusion protein remained active. Furthermore, the good detection performance of the scFv-ALP was validated through the direct competitive ELISA, and an IC_50_ of 5.85 ng/mL was obtained from the standard competitive inhibition curve (Figure 1C).

### 3.2. Characterization of MnO_2_ Nanosheets

**Figure 2:**
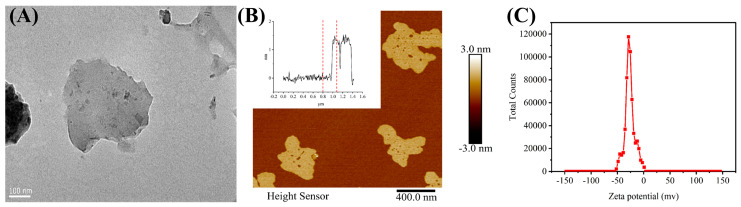
Characterization of MnO_2_ nanosheets. (**A**) TEM analysis. (**B**) AFM analysis, inset: the spectrum of nanosheet thickness analysis, and (**C**) Zeta potential test.

Due to the unique structural features of MnO_2_ nanosheets, they can serve as a peroxidase-like catalyst to oxidize TMB and induce color change. In this study, the synthesized two-dimensional nanozyme-MnO_2_ nanosheets were characterized using TEM, AFM, and a Zeta potential analyzer. As shown in Figure 2A, the TEM image reveals a typical two-dimensional sheet-like morphology with an average diameter of approximately 600–700 nm and obvious wrinkles on the surface. As shown in Figure 2B, the AFM result also indicates that the sample has an irregular sheet-like structure with a thickness of approximately 1.5 nm. Moreover, the MnO_2_ nanosheets are uniformly dispersed in the aqueous solution, which is beneficial for exposing the catalytic sites of the nanozyme and improving its catalytic reaction efficiency. Additionally, the Zeta potential test of the MnO_2_ nanosheets shows a negative Zeta potential of −25 mV, indicating that the nanosheets carry negative charges on the surface (Figure 2C). These negative charges contribute to the good dispersion and stability of the MnO_2_ nanosheets in water. In conclusion, the MnO_2_ nanosheets synthesized in this study exhibit a two-dimensional sheet-like nanostructure, good stability, and morphological characteristics. The results of the TEM, AFM, and Zeta potential tests provide important insights into the structural and physicochemical properties of the MnO_2_ nanosheets and demonstrate their potential as a highly effective enzyme mimic.

### 3.3. Feasibility Analysis and Optimization of the Nanozyme Sensing System for the scFv-ALP

**Scheme 1:**
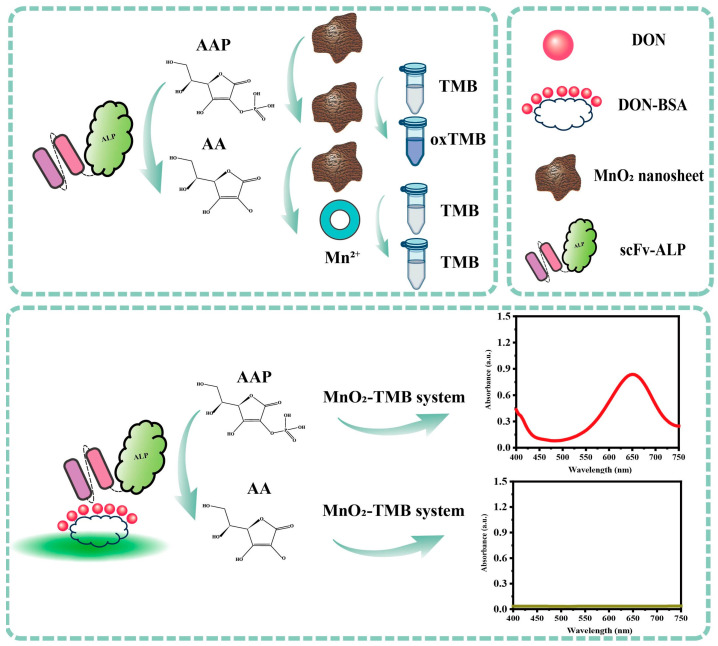
Illustration of ECAIA based on scFv-ALP and nanozyme sensing system.

**Figure 3:**
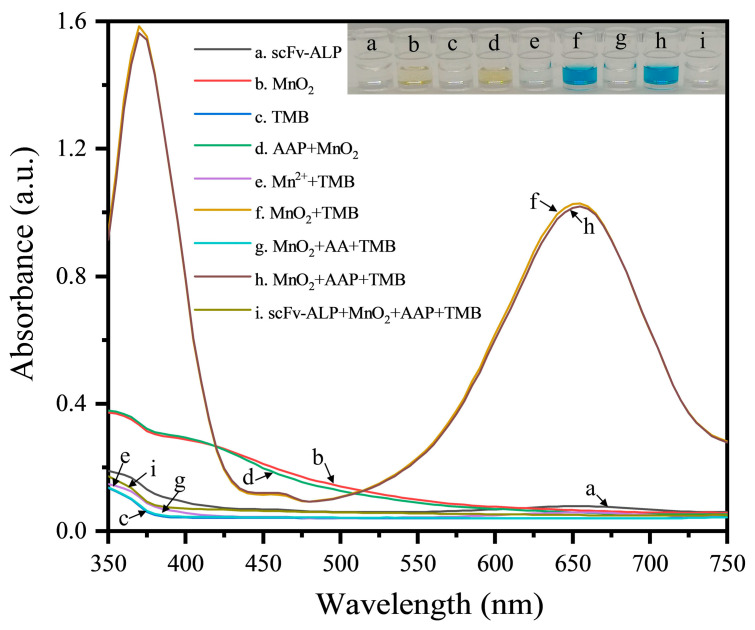
The UV–visible absorption spectra of different components in the MnO_2_-TMB system are shown. (a) scFv-ALP; (b) MnO_2_ nanosheets; (c) substrate solution AB containing TMB; (d) AAP+MnO_2_ nanosheets; (e) Mn^2+^+substrate solution AB containing TMB; (f) MnO_2_ nanosheets+substrate solution AB containing TMB; (g) MnO_2_ nanosheets+AA+substrate solution AB containing TMB; (h) MnO_2_ nanosheets+AAP+substrate solution AB containing TMB; (i) scFv-ALP+MnO_2_ nanosheets+AAP+ substrate solution AB containing TMB. Illustrations corresponding to different reaction components are presented. The components included 1 mM of MnO_2_ nanosheets, 1 mM of MnCl_2_, 1 mM of AAP, 1 mM of AA, 10 μL of 10 μg/mL scFv-ALP, 50 μL of TMB solution, 40 mM of Tris-HCl (pH 10), and 40 mM of NaAc-HAc (pH 3.8).

The principle is illustrated in Scheme 1, wherein ALP catalyzes the AAP and yields the reducing agent AA. AA, in turn, modulates the MnO_2_-TMB system. Specifically, MnO_2_ nanosheets catalyze oxidative reactions with both AA and TMB, thereby generating the detection signal. To validate the feasibility of the proposed nanozyme sensing system for detecting the scFv-ALP, a control experiment was conducted in which various combinations of reaction components within the system were tested. After the reaction, the absorbance spectra at 400–750 nm were scanned using a UV–Vis spectrophotometer, and the results are shown in Figure 3. The result indicated that in the presence of only one component (scFv-ALP, MnO_2_ nanosheets, or TMB solution), there was no significant change observed in the UV–Vis absorption spectra (curves a, b, and c). However, upon the coexistence of MnO_2_ nanosheets and TMB, the solution changed from colorless to blue, accompanied by the emergence of a prominent absorption peak at 650 nm (curve f and inset f). It was attributed to the peroxidase-like activity of MnO_2_ nanosheets, which can oxidize TMB to blue oxTMB. Furthermore, we mixed Mn^2+^ with TMB solution to evaluate the oxidation of Mn^2+^ to TMB. The result showed that there was no significant change in the absorption spectra of the system and the solution remained unchanged (inset e), indicating that Mn^2+^ did not undergo an oxidation–reduction reaction with TMB. When AA or AAP was introduced to the system, the results depicted in curves g and h of Figure 3 revealed interesting patterns. Specifically, the system containing AAP exhibited a curve similar to that observed in curve f: the solution changed from colorless to blue, accompanied by the emergence of a prominent absorption peak at 650 nm. However, when AA was added to the solution, the result was quite different. Instead of the color change, the solution remained colorless. This is because reducing AA reduced the MnO_2_ nanosheets, destroying their oxidizing properties and leaving them unable to continue catalyzing the oxidation of TMB to cause a color change in the solution. At the same time, the partially reduced AAP could not completely reduce MnO_2_ nanosheets to Mn^2+^, and the remaining MnO_2_ nanosheets oxidized TMB, resulting in a lower OD_650 nm_ than curve f (the color in inset h is slightly lighter than that in f). Based on the feasibility verification above, when the scFv-ALP, AAP, MnO_2_ nanosheets, and TMB are present in the system simultaneously, the dual-functional fusion protein scFv-ALP catalyzes the hydrolysis of AAP to generate AA, which interacts with the MnO_2_ nanosheets. As a result, the solution remains colorless and does not exhibit an absorption peak at 650 nm (curve and inset i). Therefore, the scFv-ALP and MnO_2_-TMB system can be used to develop an enzyme cascade amplification immunoassay for detecting DON. On this basis, to obtain the optimal sensing performance of the MnO2-TMB system, further optimization was performed to determine the optimal concentration of MnO_2_ nanosheets (1.4 mM) and AAP (250 μM), and the optical incubation time of 120 min for color development, as shown in Figure S1 and the Supplementary Material.

### 3.4. ECAIA for Detecting DON

**Figure 4:**
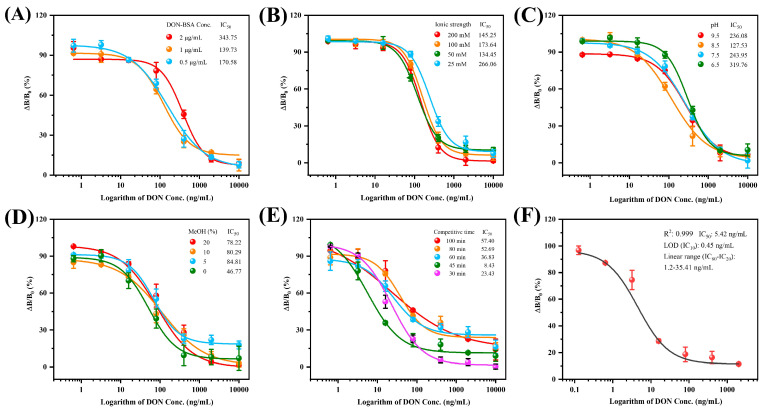
Development of the ECAIA for DON. Optimization of DON-BSA concentration (**A**), ionic strength (**B**), pH (**C**), MeOH concentration (**D**), and competitive time (**E**) for ECAIA. (**F**) The standard competitive inhibition curve of ECAIA. The error bar represents the standard deviation from three independent experiments.

To achieve optimal performance of the ECAIA for DON detection, the optimal scFv-ALP concentration (12.5 μg/mL) (Table S2) and optimal coating DON-BSA concentration (1 μg/mL) (Figure 4A) were determined using checkerboard titration and direct competition assay, respectively. Based on these results, a series of conditions such as ionic strength, pH, MeOH content in the buffer, and competition time were optimized using IC_50_ as the evaluation standard. As shown in Figure 4B, the 25 mM TBS buffer system provided the highest sensitivity (IC_50_ = 134.45 ng/mL). Similarly, the optimal reaction pH for the competition system was found to be 8.5 (IC_50_ = 127.53 ng/mL), 0% MeOH (IC_50_ = 46.77 ng/mL), and 45 min competition time (IC_50_ = 8.43 ng/mL) (Figure 4C–E).

Based on the optimized conditions, a standard curve of Log (DON concentration) vs. ΔB/B_0_ was plotted. The scatter plot was fitted with a logistic model, and the fitting equation was obtained as y = 0.11417 + [0.96659 − 0.11417]/[1 + (x/4.53891)^1.06584^] (R^2^ = 0.999). The linear range of ECAIA for DON detection was 1.2–35.41 ng/mL (IC_80_–IC_20_), and the limit of detection (LOD) was 0.45 ng/mL (IC_90_), as shown in Figure 4F. Compared to the other reported immunoassays, ECAIA exhibited a wider linear range and higher sensitivity for DON detection (Table S3), indicating its broad potential in detecting other toxic small molecules in food.

### 3.5. Selectivity of the ECAIA

The present study investigated the selectivity of ECAIA towards four mycotoxins, namely OTA, AFB_1_, FB_1_, and ZEN. Four common mycotoxins, which were used as substitutes for DON, were diluted to various concentrations (0, 0.128, 0.64, 3.2, 16, 80, 400, and 2000 ng/mL) and subsequently tested using ECAIA. The results, as shown in Table 1, indicated that the IC_50_ values for the four mycotoxins were >1200 ng/mL, and the cross-reactivity rates were <0.01%. Therefore, the ECAIA exhibits minimal cross-reactivity with those mycotoxins, suggesting good selectivity for the detection of DON.

### 3.6. Sample Analysis and Validation

Due to the wide distribution of DON in crops, the complex composition of crops can interfere with the sensitivity and accuracy of detection methods. Therefore, eliminating matrix interference is crucial for the construction of ECAIA. In this study, corn, a common cereal, was selected for the methodological evaluation of ECAIA. The results showed that a 10-fold dilution of the corn extract could directly eliminate matrix interference (Figure S2). In addition, to further validate the effectiveness of ECAIA, DON was spiked into negative corn samples at different concentrations (200, 400, and 800 μg/kg). The intra-assay recoveries of ECAIA ranged from 88.9% to 118.3%, with relative standard deviations (RSD) ranging from 2.8% to 5.2%. Additionally, the inter-assay average recoveries of ECAIA ranged from 80.1% to 109.2%, with corresponding RSD ranging from 3.5% to 10.3% (Table 2). These results indicate that ECAIA has good accuracy when applied to the detection of DON in complex matrices. Furthermore, in this study, 10 positive samples were detected using both ECAIA and HPLC-UVD. As shown in Table S4, there was no significant difference in the DON content of the same positive sample detected by ECAIA and HPLC-UVD. The detection limit for HPLC-UVD and ECAIA is 50 and 18 μg/kg, respectively, thus demonstrating that ECAIA exhibits higher sensitivity. Regression analysis of the DON content detected by ECAIA and HPLC-UVD yielded a linear equation of y = 0.94x + 14.85 (R^2^ = 0.97) (Figure S3). In summary, the developed ECAIA has good sensitivity, accuracy, and stability, and can be used for DON detection in cereals.

## 4. Conclusions

In this study, we report an enzyme cascade amplification immunoassay based on the scFv-ALP and the MnO_2_-TMB sensing system for the detection of DON in cereals. The bifunctional antibody scFv-ALP, which generates AA by hydrolysis of AAP, is used to link the antibody–antigen recognition system with the MnO_2_-TMB sensing system, thereby regulating the colorimetric system and achieving high-sensitivity detection of DON. By optimizing the relevant conditions for the MnO_2_-TMB sensing system and the competitive system, the LOD of DON in the ECAIA was achieved at 0.45 ng/mL. Given its high sensitivity and specificity in detecting DON, this immunoassay could become a standard tool for monitoring mycotoxins in cereals and other food products. Its adaptability as a detection model for other mycotoxins suggests that it could contribute significantly to reducing the risk of foodborne diseases caused by contaminated foods. With further optimization and validation, the ECAIA could become a routine analytical method in food testing laboratories worldwide. Furthermore, apart from MnO_2_ nanosheets, other novel two-dimensional nanosheets such as MoS_2_, graphitic carbon nitride, layered double hydroxides, etc., can be introduced to enhance catalytic performance. From a methodological perspective, techniques like photonic immobilization [43] and surface-enhanced Raman spectroscopy can be combined with immunoassays to improve detection sensitivity and broaden their applicability.

## Figures and Tables

**Table 1:** Cross-reactivity of ECAIA with OTA, AFB_1_, FB_1_, and ZEN.

Analyte	Chemical Structure	IC_50_	CR (%)
DON	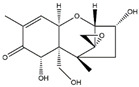	5.62	100
OTA	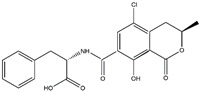	>1200	<0.01
AFB_1_	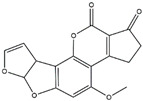	>1200	<0.01
FB_1_	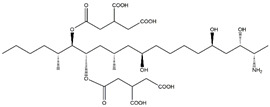	>1200	<0.01
ZEN	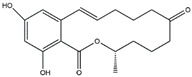	>1200	<0.01

**Table 2:** Recoveries of DON from the spiked cereals samples determined by ECAIA.

Test	Spiked DON (μg/kg)	Mean ± SD (μg/kg)	Recovery (%)	RSD (%)
Intra-assay (*n* = 3) ^a^	200	236.54 ± 6.67	118.3	2.8
	400	384.71 ± 19.32	96.2	5
	800	711.27 ± 36.78	88.9	5.2
Inter-assay (*n* = 3) ^b^	200	218.34 ± 22.43	109.2	10.3
	400	320.46 ± 23.96	80.1	7.5
	800	699.76 ± 24.62	87.5	3.5

^a^ Each assay was performed in triplicate on the same day. ^b^ Each assay was performed in triplicate for three days.

## Data Availability

The original contributions presented in the study are included in the article/Supplementary Materials, further inquiries can be directed to the corresponding authors.

## Supplementary Materials

The following supporting information can be downloaded at: https://www.mdpi.com/article/10.3390/foods13132009/s1, Figure S1: Optimization of the MnO_2_-TMB system; Figure S2: Evaluation of the corn matrix effect on ECAIA; Figure S3: Linear regression analysis of the detection results by ECAIA and HPLC-UVD; Table S1: Parameters of HPLC-UVD; Table S2: Optimization of scFv-ALP concentration by checkerboard method; Table S3: Comparison of the developed ECAIA with the other reported immunoassays for DON; Table S4: Detection of DON content in positive corn samples by HPLC-UVD and ECAIA. References [44,45,46] are cited in the Supplementary Materials.
