# Supplementary material for: Enzyme Cascade Amplification-Based Immunoassay Using Alkaline Phosphatase-Linked Single-Chain Variable Fragment Fusion Tracer and MnO2 Nanosheets for Detection of Deoxynivalenol in Corn Samples
Source: Foods. 2024 Jun 25;13(13):2009. doi: 10.3390/foods13132009 (PMC11241725; doi:10.3390/foods13132009)
Supplement: Supplementary file 1 [file foods-13-02009-s001.zip › foods-3024824-supplementary.pdf]

Supplementary Material

Enzyme Cascade Amplification-Based Immunoassay Using Alkaline Phosphatase-Linked Single-Chain Variable Fragment Fusion Tracer and MnO_2 Nanosheets for Detection of Deoxynivalenol in Corn Samples

Guifang Xie [a, c, 1], Fujing Mao [a, 1], Yirui Huang [a], Li Wen [a], Zhichang Sun [a], Zhenyun He [b,*], Xing Liu [a,*]

[a] *School of Food Science and Engineering, Hainan University, Haikou 570228, China*

[b] *Hainan College of Economics and Business, Haikou 571127, China*

[c] *Guizhou Provincial Supervision and Testing Center for Agricultural Product Quality, Agricultural Product Quality and Safety Risk Assessment Laboratory of the Ministry of Agriculture, Guiyang 550004, China*

* Corresponding author.

Prof. Dr. Xing Liu, E-mail address: xliu@hainanu.edu.cn

Dr. Zhenyun He, E-mail address: zhenyun89@foxmail.com

[1] These authors contributed equally to this work

Contents

Pretreatment of corn samples for HPLC-UVD--S-3

Table S1. Parameters of HPLC-UVD--S-4

Optimization of the MnO_2-TMB system--S-5

Figure S1. Optimization of the MnO_2-TMB system-----------------------------------S-6

Table S2. Optimization of scFv-ALP concentration by checkerboard method------S-7

Table S3. Comparison of the developed ECAIA with the other reported immunoassays for DON--S-8

Figure S2. Evaluation of the corn matrix effect on ECAIA----------------------------S-9

Figure S3. Linear regression analysis of the detection results by ECAIA and HPLC-UVD---S-10

Table S4. Detection of DON content in positive corn samples by HPLC-UVD and ECAIA---S-11

References--S-12

Pretreatment of corn samples for HPLC-UVD

Accurately weigh 5.0 g of ground corn sample into a centrifuge tube, followed by the addition of 1 g of polyethylene glycol and 20 mL of distilled water. The mixture is subjected to extraction on a shaker for 20 min, followed by centrifugation (10,000×*g*, 10 min). The supernatant is transferred to a clean centrifuge tube and then extracted using an HLB solid-phase extraction column (Jiangsu Green Union Science Instrument Co. Ltd., Taizhou, China), followed by elution with 4 mL of methanol and drying under nitrogen gas. Residues are dissolved in 1 mL of methanol and filtered through a 0.22 μm membrane filter. The filtrate is collected for analysis by HPLC-UVD.

Table S1. Parameters of HPLC-UVD

Parameter names	Parameter values[a]
Chromatographic column	C18 column with 150 mm length, 4.6 mm inner diameter, and 5 µm particle size
Mobile phase	methanol–water (20:80, v/v);
Column temperature	35 °C
Flow rates	0.8 mL/min
Injection volume	10 µL
Detection wavelength	218 nm

[a] This parameter refers to the Chinese standard (GB5009.111-2016).

Optimization of the MnO_2-TMB system

To obtain the best sensing performance of the MnO_2-TMB system, this study optimized three parameters, including the concentration of MnO_2 nanosheets, the concentration of AAP, and the incubation time for color development. Firstly, as shown in Figure S1A, different concentrations of MnO_2 nanosheets (0.4, 0.6, 0.8, 1, 1.2, 1.4, 1.6, 1.8, and 2 mM) were added to the system. The $\Delta A_{650\ nm}$ of the solution increased with the concentration of nanosheets and became flat at 1.4 mM, indicating that 1.4 mM of MnO_2 nanosheets reached saturation in the system. Therefore, 1.4 mM of MnO_2 nanosheets were selected for subsequent experiments. Considering the significant effect of AAP on the MnO_2-TMB sensing system, a series of AAP concentrations (3.90, 7.81, 15.63, 31.25, 62.5, 125, 250, 500, and 1000 μM) were added to the system for optimization. As shown in Figure S1B, the $\Delta A_{650\ nm}$ increased with increasing AAP concentration and reached a maximum at 250 μM of AAP, indicating that the optimal AAP concentration for detection was 250 μM. Furthermore, the effect of color development time on the MnO_2-TMB system was investigated. The $\Delta A_{650\ nm}$ increased with time and became stable after 120 min, indicating that the components in the system reacted completely and reached a stable state. Therefore, 120 min was determined to be the optimal color development time for the MnO_2-TMB sensing system (Figure S1C). In summary, 1.4 mM of MnO_2 nanosheets, 250 μM of AAP, and 120 min of color development time were selected as the optimal parameters for the MnO_2-TMB sensing system, and an ECAIA for detecting DON was constructed based on these parameters.

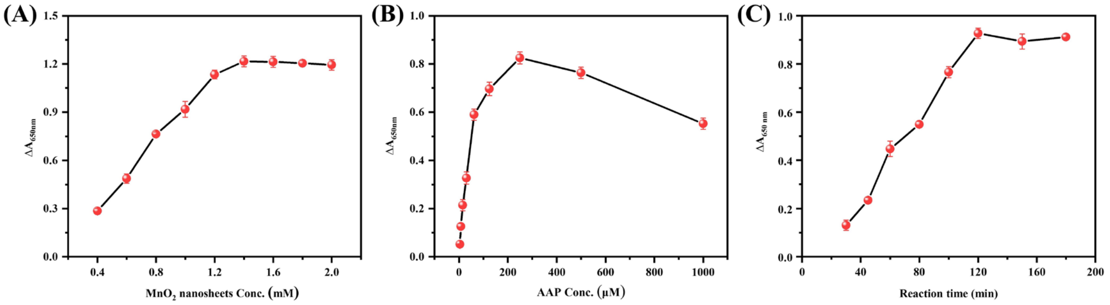

Figure S1. Optimization of the MnO_2-TMB system. (A) Optimization of MnO_2 nanosheets concentration. The tested concentrations of MnO_2 nanosheets include 0.4, 0.6, 0.8, 1, 1.2, 1.4, 1.6, 1.8, and 2 mM. (B) Optimization of AAP concentration. The tested AAP concentrations include 3.90, 7.81, 15.63, 31.25, 62.5, 125, 250, 500, and 1000 μM. (C) Incubation time for reaction in the system. The tested incubation times include 30, 45, 60, 80, 100, 120, 150, and 180 min. The error bar represents the standard deviation from three independent experiments.

Table S2. Optimization of scFv-ALP concentration by checkerboard titration.

OD_{405}		DON-BSA (μg/mL)			
		2	1	0.5	0.25
scFv-ALP (μg/mL)	3.125	0.315	0.309	0.289	0.327
	6.25	0.327	0.345	0.374	0.346
	12.5	0.351	0.358	0.395	0.362
	25	0.294	0.301	0.37	0.255
	50	0.254	0.252	0.285	0.265

Table S3. Comparison of the developed ECAIA with the other reported immunoassays for DON.

Method	Material	Linear range (ng/mL)	LOD (ng/mL)	Refs.
Magnetic immunoassay	mAb, Au NBPs, MBs	0–2000	57.93	[1]
LFIA	mAb, GNPs	3.46–43.64	3.46	[2]
LFIA	mAb, QDNBs,	0.465–16.19	2.97	[3]
pELISA	mAb, PDA NPs, Tyramine, HRP-SA	1–1000	0.435	[4]
ELISA	nanobody	2.18–62.25	1.16	[5]
ECAIA	scFv-ALP, MnO_2 nanosheets	1.2–35.41	0.45	This work

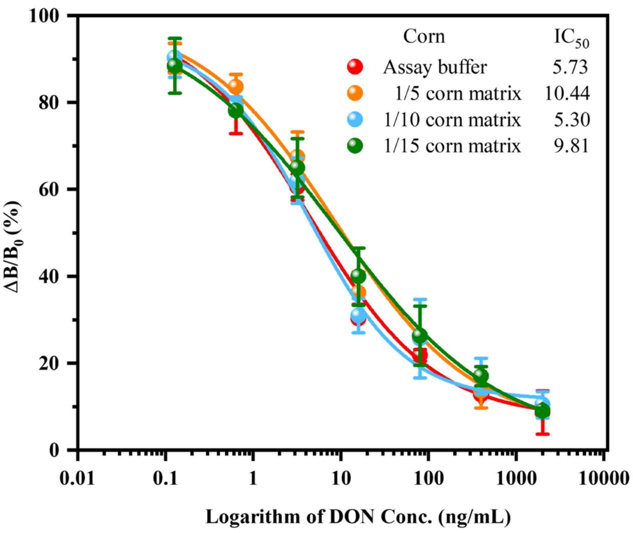

Figure S2. Evaluation of the corn matrix effect on ECAIA. The error bar represents the standard deviation from three independent experiments.

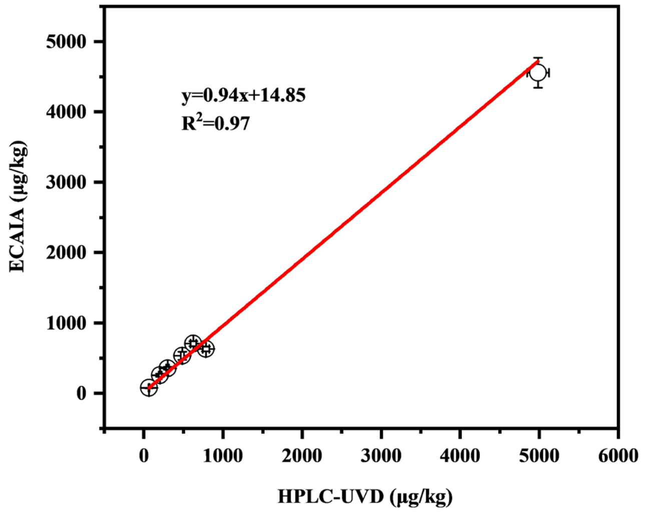

Figure S3. Linear regression analysis of the detection results by ECAIA and HPLC-UVD. The error bar represents the standard deviation from three independent experiments.

Table S4. Detection of DON content in positive corn samples by HPLC-UVD and ECAIA.

Sample	HPLC-UVD (μg/kg, n=3)		ECAIA (μg/kg, n=3)	
	Mean ± SD	RSD (%)	Mean ± SD	RSD (%)
C1	63.75 ± 6.19	9.7	73.56 ± 2.59	3.5
C2	4983.22 ± 137.30	2.8	4556.54 ± 213.82	4.7
C3	782.76 ± 41.10	5.3	629.41 ± 32,21	5.1
C4	486.37 ± 21.63	4.4	531.42 ± 57.94	10.9
C5	300.81 ± 17.97	6.0	354.78 ± 19.03	5.4
C6	ND[a]	-	39.33 ± 1.79	4.6
C7	250.16 ± 19.37	9.4	252.90 ± 23.35	9.2
C8	ND	-	50.21 ± 3.41	6.8
C9	ND	-	28.37 ± 2.49	8.8
C10	622.03 ± 35.94	5.8	704.19 ± 34.54	4.9

[a] Not detected.

References

1. Guo, R.; Ji, Y.; Chen, J.; Ye, J.; Ni, B.; Li, L.; Yang, Y. Multicolor Visual Detection of Deoxynivalenol in Grain Based on Magnetic Immunoassay and Enzymatic Etching of Plasmonic Gold Nanobipyramids. *Toxins* **2023,** *15* (6), 351–361.
2. Feng, J.; Xue, Y.; Wang, X.; Song, Q.; Wang, B.; Ren, X.; Zhang, L.; Liu, Z. Sensitive, simultaneous and quantitative detection of deoxynivalenol and fumonisin B(1) in the water environment using lateral flow immunoassay integrated with smartphone. *Sci. Total Environ.* **2022,** *834*, 155354–155361.
3. Hou, S.; Ma, J.; Cheng, Y.; Wang, H.; Sun, J.; Yan, Y. Quantum dot nanobead-based fluorescent immunochromatographic assay for simultaneous quantitative detection of fumonisin B1, dexyonivalenol, and zearalenone in grains. *Food Control* **2020,** *117*, 107331–107339.
4. Hong, F.; Chen, R.; Lu, P.; Li, L.; Xiao, R.; Chen, Y.; Yang, H. A universal, portable, and ultra-sensitive pipet immunoassay platform for deoxynivalenol detection based on dopamine self-polymerization-mediated bioconjugation and signal amplification. *J. Hazard. Mater.* **2022,** *436*, 129257–129265.
5. Qiu, Y.L.; He, Q.H.; Xu, Y.; Bhunia, A.K.; Tu, Z.; Chen, B.; Liu, Y.Y. Deoxynivalenol-mimic nanobody isolated from a naive phage display nanobody library and its application in immunoassay. *Anal. Chim. Acta* **2015,** *887*, 201–208.
